# Low‐dose 2.5 MV cone‐beam computed tomography with thick CsI flat‐panel imager

**DOI:** 10.1120/jacmp.v17i4.6185

**Published:** 2016-07-08

**Authors:** Grace Tang, Christopher Moussot, Daniel Morf, Edward Seppi, Howard Amols

**Affiliations:** ^1^ Department of Medical Physics Memorial Sloan Kettering Cancer Center New York NY USA; ^2^ Varian Medical Systems Cranston NJ USA; ^3^ Varian Medical Systems iLab GmbH Baden‐Dättwil Switzerland; ^4^ Varian Medical Systems, Ginzton Technology Center Palo Alto CA USA

**Keywords:** low‐dose megavoltage cone‐beam, thick scintillator, high‐efficiency imager

## Abstract

Most of the treatment units, both new and old models, are equipped with a megavoltage portal imager but its use for volumetric imaging is limited. This is mainly due to the poor image quality produced by the high‐energy treatment beam (>6 MV). A linac at our center is equipped with a prototype 2.5 MV imaging beam. This study evaluates the feasibility of low‐dose megavoltage cone‐beam imaging with the 2.5 MV beam and a thick cesium iodide detector, which is a high‐efficiency imager. Basic imaging properties such as spatial resolution and modulation transfer function were assessed for the 2.5 MV prototype imaging system. For image quality and imaging dose, a series of megavoltage cone‐beam scans were acquired for the head, thorax, and pelvis of an anthropomorphic phantom and were compared to kilovoltage cone‐beam and 6X megavoltage cone‐beam images. To demonstrate the advantage of MV imaging, a phantom with metallic inserts was scanned and the image quality was compared to CT and kilovoltage cone‐beam scans. With a lower energy beam and higher detector efficiency, the 2.5 MV imaging system generally yields better image quality than does the 6 MV imaging system with the conventional MV imager. In particular, with the anthropomorphic phantom studies, the contrast to noise of bone to tissue is generally improved in the 2.5 MV images compared to 6 MV. With an image quality sufficient for bony alignment, the imaging dose for 2.5 MV cone‐beam images is 2.4−3.4 MU compared to 26 MU in 6 MV cone‐beam scans for the head, thorax, and pelvis regions of the phantom. Unlike kilovoltage cone‐beam, the 2.5 MV imaging system does not suffer from high‐Z image artifacts. This can be very useful for treatment planning in cases where high‐Z prostheses are present.

PACS number(s): 87.57.Q‐

## I. INTRODUCTION

With the continual development of image‐guided radiation therapy (IGRT) and the increasing popularity of stereotactic and hypofractionation treatments, the use of kilovoltage cone‐beam computed tomography (kVCB) for accurate patient setup and localization has vastly increased in the past decade. Although kV/kV or kV/MV planar image pairs are sufficient for bony alignment, 3D kVCB systems are often capable of facilitating the ability to register images with soft

tissues as well. More importantly, these kV imaging systems are fully integrated to treatment units and are readily available. However, kV image quality can be significantly perturbed by high‐Z materials, especially for patients with metallic implants such as dental fillings, hip prosthesis, and prostate seeds. These high‐Z image artifacts are greatly reduced in MV X‐ray imaging. Past efforts of using the therapeutic MV beams for imaging have proven its feasibility especially in the area of portal imaging while the implementation of megavoltage cone‐beam imaging (MVCB) has also been studied by several different groups^1^, ^2^, ^3^, ^4^, ^5^, ^6^, ^7^, ^8^, ^9^ and is commercially available (MVision, Siemens Medical Solutions USA, Malvern, PA).

Since most linacs are already equipped with a MV portal imager, one would expect that MVCB can be readily implemented in the clinic with minimal additional cost or modification to the treatment units. Unfortunately, MVCB has yet to be widely implemented, in part because high‐energy photons from the therapeutic MV beams (typically 6 MV) often produce images with poor tissue contrast and the imaging doses are relatively high. While this can be resolved by lowering the energy of the MV beam, it can be mechanically challenging to produce a stable and sufficiently low‐energy MV beam that attains similar imaging quality to kV X‐rays for current waveguide designs. The lowest energy beam for portal imaging in a modern linac of 3.5−4 MV has been reported^7^, ^8^, ^10^ with the use of thin low‐Z target, which was first proposed by Galbraith in 1989.^(11)^ A low‐Z target increases the portion of low‐energy photons in the output spectrum due to the decrease in photoelectric absorption in low‐Z material. The thinner thickness of the target also decreases the amount of self‐absorption and any further attenuation of these low‐energy photons can be eliminated by removing the flattening filter. This beamline configuration subsequently improves the image contrast as demonstrated by several previous studies.^10^, ^12^, ^13^, ^14^, ^15^

A new TrueBeam (Varian Medical Systems, Palo Alto, CA) at our center is equipped with a prototype 2.5 MV unflattened imaging beam. To further improve imaging efficiency, a prototype portal imager consisting of an 8 mm–thick cesium iodide (CsI) scintillator is also being tested. This study demonstrates the feasibility and image quality of low‐dose MVCB using 2.5 MV X‐rays with a highly efficient CsI detector.

## II. MATERIALS AND METHODS

### A. Prototype MV imaging system in TrueBeam

**Figure 1:**
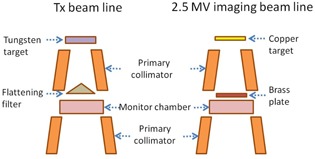
Schematics of the 2.5 MV imaging beamline in comparison to the treatment beamline in the treatment head (not to scale).

A nonclinical prototype MV imaging system was installed on a Varian TrueBeam linac. Both hardware and software controls were fully integrated into the linac control console. The configuration of the MV imaging beamline is similar to the treatment beamline but the imaging beamline uses a thin copper target without a flattening filter, as illustrated in Fig. 1. The 2.5 MV beam is generated with a 2.5 MeV monoenergetic electron beam incident on a 2 mm–thick copper target. In place of a flattening filter, a 0.81 mm‐thick brass plate is used as the port cover on the rotatable carousel. Downstream the brass plate, the unflattened 2.5 MV beam traverses the rest of the treatment head components such as monitor chambers and jaws before exiting the Mylar glass window. The allowable dose rates of this low‐energy MV imaging beam are 5, 10, 20, 30, and 50 MU/min. The output of the 2.5 MV beam is nominally calibrated to 1 cGy/MU at 0.5 cm depth in water for a $10\times 10\;{\text{cm}}^{2}$ field.

Another key component of this prototype MV imaging system is the high‐efficiency CsI detector, comprised of an 8 mm–thick pixelated thallium‐doped CsI scintillation layer coupled to an array of amorphous silicon thin film transistors (TFTs). The active area of the crystal layer is $38.8\times 28.5\;{\text{cm}}^{2}$ with a pixel size of $0.38\times 0.38\;{\text{mm}}^{2}$. The TFTs array is the same as that in the aS1000 portal imager model (Portal Vision, Varian Medical Systems), which has a pixel matrix of 2048×1536. However, the panel was operated at a 2×2 bin mode, resulting in an effective pixel matrix of 1024×769 for all the images acquired in this study (i.e., with an effective pixel size of $0.38\times 0.38\;{\text{mm}}^{2}$). The electronic operation control of both detectors is the same with a maximum frame rate of 9 frames per second and a readout time of 100 μs. Details of the construction of this high‐efficiency detector can be found in Seppi et al.^(16)^ Compared to the conventional aS1000 model, which consists of a 0.4 mm–thick layer of gadolinium oxysulfide scintillator, the CsI detector has an 8 mm–thick CsI crystal, which has a higher X‐ray absorption efficiency.^(16)^ Hence, the imaging dose is expected to be reduced with the thick CsI detector. To evaluate the overall performance and efficiency of the system, image quality and imaging dose of MVCB using the prototype system (i.e., 2.5 MV beam with CsI detector) was compared to the conventional MV imaging system (i.e., 6 MV beam with aS1000).

### B. Image acquisition

MVCB scans were acquired in continuous mode where individual frames were read out between beam pulses as the linac performed a short “beam hold”. The number of beam pulses per frame or the dose per frame (DPF) is defined by the user, using Varian proprietary XML commands in the Developer mode of TrueBeam. The minimum dose per beam pulse for the 2.5 MV beam is 0.0025 MU, but the minimum DPF used in the MVCB scans was 0.005 MU (i.e., 2 beam pulses per frame) to improve signal‐to‐noise ratio (SNR). Regardless of the DPF, the gantry rotates at its maximum speed of approximately 1 revolution per minute. Thus, the number of projections in a MVCB scan can only be controlled by the dose rate for a given DPF configuration. To demonstrate the variation of image quality and imaging dose, two sets of 2.5X MVCB scans were acquired using two different DPF settings (i.e., low‐dose and high‐dose). A different dose rate was used in each set of the images, such that an average of 450 projections was collected in each 360° scan. Calibration procedures for the CsI detector are similar to those for the conventional imager and are performed online. Calibration images, such as the dark field, flood field, and pixel defect map, were obtained to facilitate any electronic offsets and field uniformity corrections to the raw projections. In addition, isocenter offset was corrected by IsoCal, which is the isocenter calibration procedure for cone‐beam computed tomography (CBCT) modality on TrueBeam. All raw projections were exported and reconstructed offline using research software based on the Feldkamp back‐projection algorithm^(17)^ (iTools, Varian Medical Systems iLab GmbH, Baden‐Dättwil, Switzerland).

A series of MVCB phantom scans were acquired of the RANDO phantom (Radiology Support Devices, Long Beach, CA) at a source to imager distance (SID) of 150 cm with a field size of $26\times 20\;{\text{cm}}^{2}$. Depending on the size of the scanning object, either the full‐fan scanning mode or the half‐fan scanning mode was used. For half‐fan scanning, there was a lateral offset of the detector (typically 6−8 cm) to increase the field‐of‐view; that is to say, the thorax and pelvis regions of the RANDO phantom were scanned in the half‐fan setting while the head region was scanned with a full‐fan setting (i.e., no detector offset). Different from kVCB, both full‐fan and half‐fan MVCB images were scanned with a full 360° rotation. The image quality of the 2.5X MVCB scans was assessed and compared to that of 6X MVCB and kVCB. The DPF used for

6X MVCB was chosen such that the resultant image quality matched the low‐dose 2.5X MVCB imageset. 6 MV and 2.5 MV projections were exported from the linac and 3D images were reconstructed offline. However, for kVCB, all images were acquired with the typical clinical settings and reconstructed online using the conventional kV imaging system that is integrated into the linac (On‐board Imager, Varian Medical Systems). To further investigate the imaging properties of the 2.5 MV X‐rays, scans were acquired of a polymethyl methacrylate (PMMA) phantom with different inserts of air, brass, aluminum, and steel. Image artifacts of the high‐Z materials were compared to the corresponding kVCB scans and kV fan beam (kVFB) scans, which are typically used in patient simulation.

### C. Imaging dose

Doses for 2.5X MVCB were measured in a computed tomography dose index (CTDI) head and body phantom (CSP Medical, London, Ontario, Canada) using a 0.6 cc Farmer chamber (Exradin A12, Standard Imaging, Middleton, WI). Based on AAPM‐TG51,^(18^) the 2.5X MVCB dose to water $D_{W}^{2.5MV}$ is:

(1) $$D_{W}^{2.5MV}=M\times N_{D,w}^{60_{Co}}$$

where *M* is the corrected measurement reading, and $N_{D,w}^{60_{Co}}$ is the dose calibration factor. The beam quality of the 2.5 MV X‐rays is similar to a cobalt 60 beam with a $d_{\text{max}}$ of 0.5 mm. From a $10\times 10\;{\text{cm}}^{2}$ depth‐dose measurement in water, it was found that the %dd(10) for the 2.5 MV imaging beam is 52.0%. Extrapolating the plot for Exradin A12 chamber in Fig. 4 of the TG‐51 protocol, the $k_{Q}$ for the 2.5 MV beam is expected to be within 1% from unity (c.f. $k_{Q}$ for 6 MV beam is 0.996, while the %dd(10) of 6 MV beam is 66.0% and 58.0% for cobalt). Therefore, the kQ for the 2.5 MV beam is assumed to be 1 in the dose measurements.

The CTDI head phantom has a radius of 8 cm and dose was measured at the isocenter and 1 cm below surface. Two different sets of measurements were performed with a full‐fan scan using 3 MU and 8 MU. Similar measurements were taken for the CTDI body phantom, which has a radius of 16 cm, using the half‐fan scan setting. Dose was measured at the isocenter and 1 cm below surface for two sets of MVCB scan using 8 MU and 14 MU. The same measurements were repeated for kVCB but using the standard clinical acquisition parameters and a kV chamber (Accu Pro and Accu kV, Radcal Corporation, Monrovia, CA).

### D. Image resolution

Spatial resolution was quantified by determining the modulation transfer function (MTF) of the prototype system. Planar images of a spatial resolution phantom were acquired. The spatial resolution phantom consists of six different brass inserts with line‐pair patterns of 1.000, 0.500, 0.333, 0.250, 0.167, and 0.125 lp/mm embedded in a slab of Lucite. The phantom was placed directly on top of the detector, which was located at SID 150 cm. Each insert was irradiated separately by shifting the center of the insert to the isocenter. A high‐resolution projection (1024×768) was acquired for each insert with 3 MU. For comparison, the same measurements were repeated for the conventional 6 MV imaging system. For contrast resolution, the contrast‐to‐noise ratios (CNRs) between tissue and bones were quantified for the RANDO phantom scans and were compared between 2.5X MVCB, 6X MVCB, and kVCB. Furthermore, both contrast and spatial resolution were qualitatively measured using a contrast detail phantom (Las Vegas Phantom, Varian Medical Systems).

## III. RESULTS

### A. System characteristics

**Figure 2:**
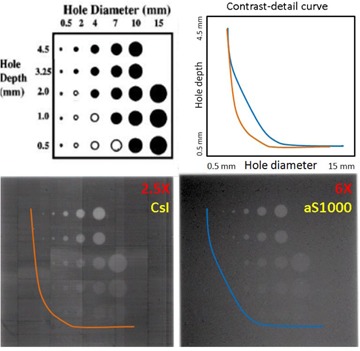
Contrast‐detail curves of 2.5X/CsI and 6X/aS1000 systems.

The contrast resolving power of the 2.5X/CsI and 6X/aS1000 systems were subjectively investigated using a contrast‐detail phantom, which has a series of holes with different size and depths for detail and contrast tests, respectively. Contrast‐detail curves were generated qualitatively based on the projection images of the phantom that were acquired with 1 MU. As shown in Fig. 2, the 2.5X/CsI system is better able to resolve smaller and lower‐contrast objects than the 6X/aS1000 system because of the lower energy of the imaging beam and the higher efficiency of the image detector.

**Figure 3:**
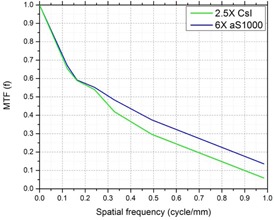
MTF of 2.5X MVCB and 6X MVCB systems.

Spatial resolution was quantified by the MTF, which was measured using an in‐house phantom. The phantom consists of multiple brass inserts that have different bar patterns: 0.125 lp/ mm, 0.167 lp/mm, 0.250 lp/mm, 0.333 lp/mm, 0.500 lp/mm, and 1.000 lp/mm. The MTF indicates that the 8 mm–thick scintillation crystal layer in the CsI panel degrades the spatial resolution compared to aS1000, which has a much thinner scintillation layer, as shown in Fig. 3. For smaller objects that are of spatial frequency higher than approximately 0.35 cycle/mm, the resolution is decreased by about 25% with the CsI detector.

### B. Phantom studies

**Figure 4:**
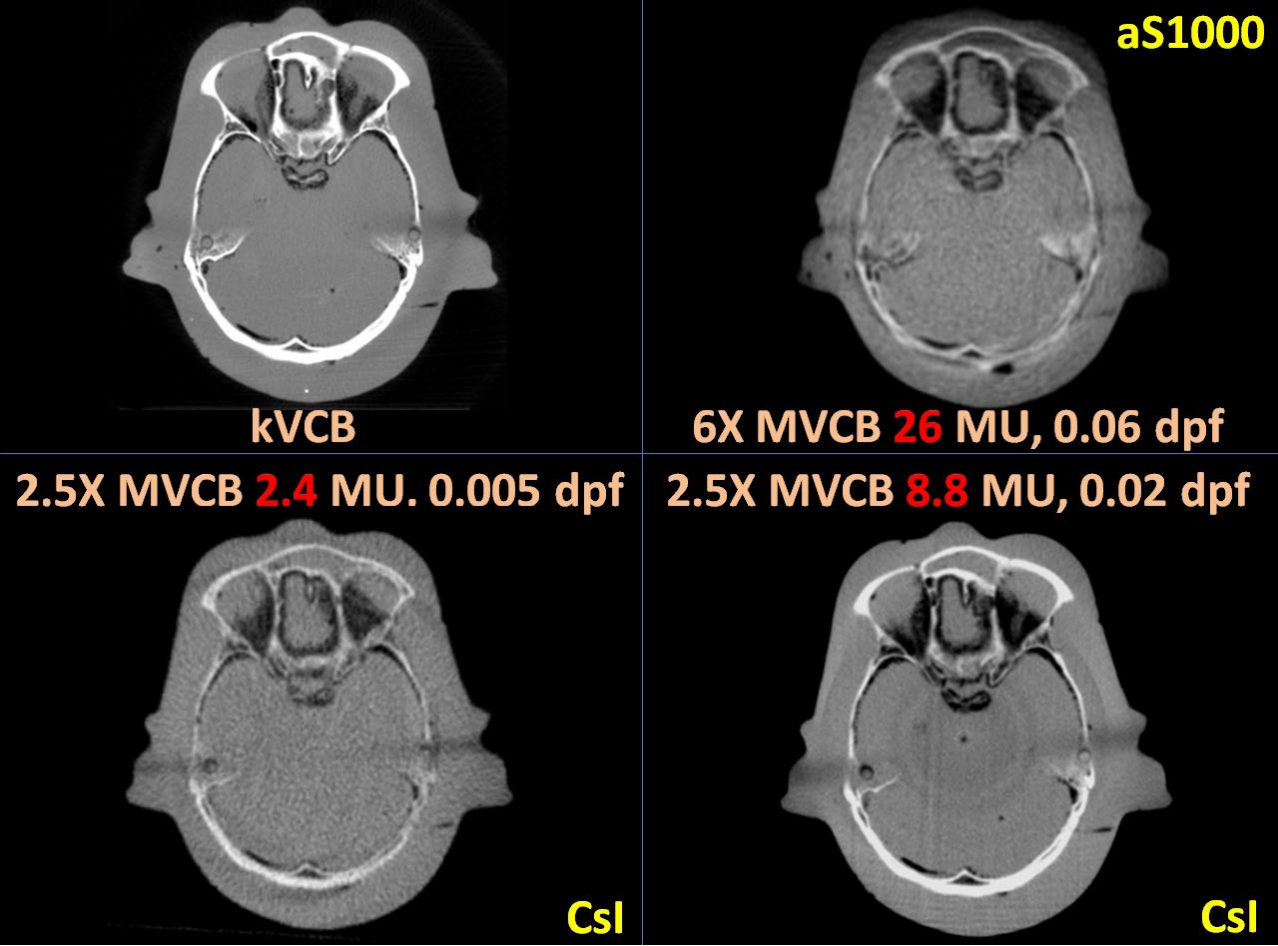
CBCT scans of the RANDO head phantom.

**Figure 5:**
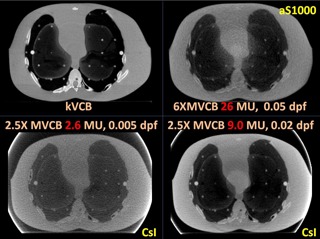
CBCT scans of the RANDO thorax phantom.

**Figure 6:**
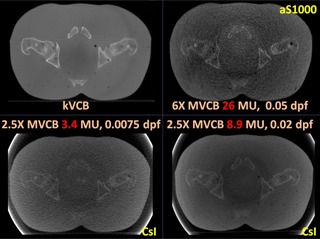
CBCT scans of the RANDO pelvis phantom.

4, 5, 6, 7 show the CBCT comparisons for the head, thorax, and pelvis phantoms, respectively. In general, 2.5X MVCB system requires approximately 10 times less dose than the 6X MVCB system to achieve sufficient image quality for patient setup purposes using bony alignment. This is due to several factors: 1) higher efficiency of CsI detector, 2) higher absorption coefficient of 2.5 MV beam, and 3) higher photoelectric cross section for 2.5 MV X‐rays. As shown in Fig. 4, the 6X MVCB scan was acquired with 26 MU while only 2.4 MU was used in the corresponding 2.5X MVCB scan. Although these low doses of 2.5X MVCB scans were sufficient for bony alignment, comparable tissue contrast to kVCB can only be achieved by increasing the dose to approximately 9 MU. By qualitative observation, the 2.5X MVCB images do not show any blurring, due to the thick CsI detector. On the other hand, some blurring was observed in the 6X MVCB images owing to the ring artifact correction applied during reconstruction. Similar results were seen in the thorax and pelvis scans, which are shown in Figs. 5 and 6. To quantify the image quality of the CBCT images, CNR was calculated as:

(2) $$CNR=\frac{\left|{\bar{I}}_{tissue}-{\bar{I}}_{bone}\right|}{\sigma_{tissue}}$$

where ${\bar{I}}_{tissue}$ and ${\bar{I}}_{bone}$ are the mean pixel values of regions of interest (ROIs) in tissue and bone, respectively, and $\sigma_{tissue}$ is the standard deviation or noise of the ROI in tissue. The CNR of bone to tissue for all scans are given in Table 1. Compared to 6X MVCB, CNR of bone to tissue is superior for 2.5X MVCB scans. However, for the pelvic region, the low‐dose 2.5X MVCB scan has lower CNR than the 6X MVCB scan. This might be due to both the increase in scatter (i.e., increased size of scanning region) and decrease in SNR (i.e., low dose used) in the 2.5X MVCB images. The increased noise in the 2.5X MVCB scan may be introduced by the imperfection of the scintillation crystal pixelation and calibration correction. As an example, this effect can be observed in Fig. 2, where the 2.5X image embeds the crystal structure of the CsI panel while this is not seen in the 6X image that was acquired with aS1000. Nonetheless, with less than 3.4 MU, 2.5X MVCB yields acceptable images for bony registration in patient setup.

**Figure 7:**
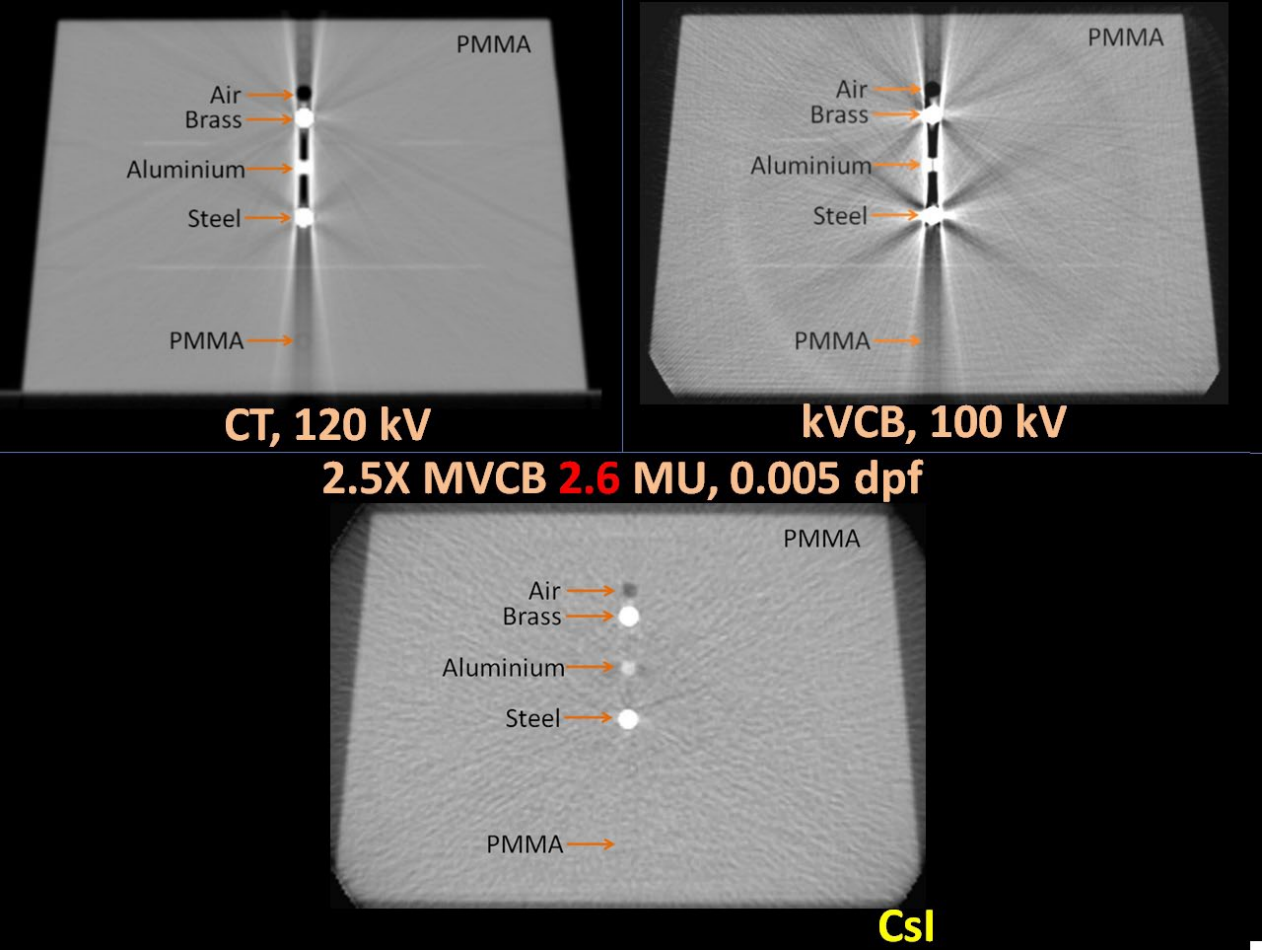
Fan‐beam CT (kVFB), kVCB, and 2.5X MVCB of a PMMA phantom with metallic inserts.

Another advantage of the 2.5X imaging system is the reduction in high‐Z image artifacts, as shown in Fig. 7, where a kVFB, kVCB, and a 2.5X MVCB scan of a PMMA phantom with inserts of air, brass, aluminum, and steel were compared. While the kV images show significant artifacts, the image quality of 2.5X MVCB was not affected by these high‐Z materials.

**Table 1:** CNR of different tissues and regions of RANDO for 2.5X MVCB, 6X MVCB, and kVCB. For the low dose 2.5X MVCB scan, 2.4 MU, 2.6 MU, and 3.4 MU were used for the head, thorax, and pelvis, respectively; while for the high dose 2.5X MVCB scan, 8.8 MU, 9.0 MU, and 8.9 MU were used, respectively. For 6X MVCB, 26 MU were used for all three phantom regions

		*Contrast‐to‐Noise Ratio (CNR)*			
*Phantom/Region*	*2.5X MVCB low dose*	*2.5X MVCB high dose*	*6X MVCB*	*kVCB*
RANDO Head	Bone – Tissue	11.6	30.3	4.7	49.5
RANDO Thorax	Bone – Tissue Bone – Lung	3.5 7.3	3.3 22.1	2.6 12.3	18.1 69.5
RANDO Pelvis	Bone – Tissue	2.9	7.4	5.4	39.5

### C. MVCB dose

**Figure 8:**
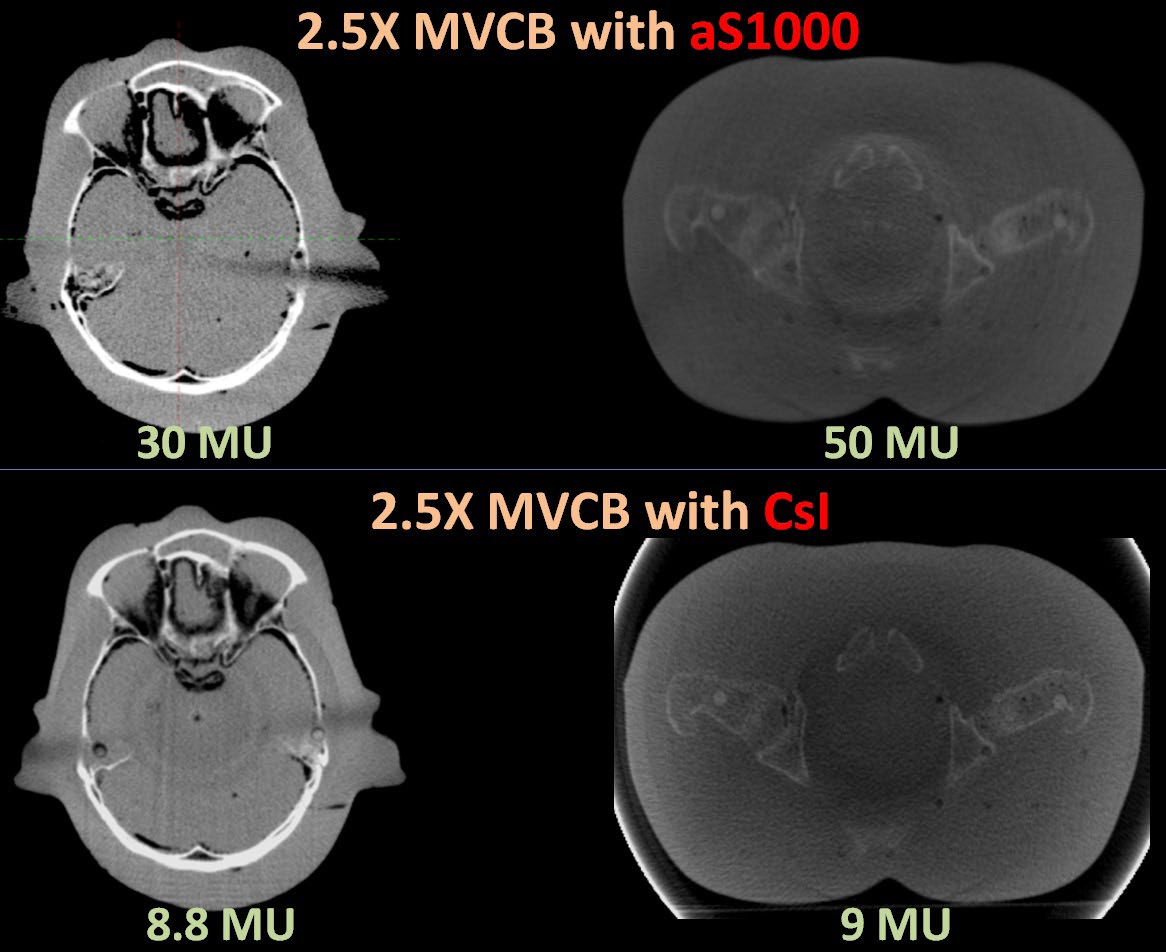
For similar image quality, the imaging dose of MVCB with CsI imager is approximately 3–5 times less than MVCB with aS1000, as demonstrated in a head scan and a pelvis scan.

Intuitively, the use of a high efficiency detector can effectively reduce the imaging dose. With the same beam energy, the dose required to achieve the same image quality is approximately five times lower when the CsI panel is used instead of the aS1000 detector. This is demonstrated in Fig. 8, where 30 MU and 50 MU was used in 2.5X MVCB scan with aS1000 for a head and pelvis phantom, respectively, while only 9 MU was used with the CsI panel.

The absolute imaging dose was measured for the 2.5X MVCB imaging system with CsI detector using the CTDI phantoms. Table 2 tabulates the point doses per MU at isocenter and 1 cm below surface for the high‐ and low‐dose 2.5X MVCB scans. As a reference, the imaging dose for kVCB was also measured and is tabulated as the total dose of the scan. The range of kVCB dose for the body phantom represents the imaging dose from different scan techniques. MVCB dose is listed in cGy per MU and the nominal total dose is stated in cGy, which is calculated based on the low‐dose scan setting for the head and body phantoms. For a low‐dose 2.4 MU MVCB head scan with a full‐fan scan setting, the absolute imaging dose at the isocenter is approximately 2.4 MU×0.56cGy/MU=1.34 cGy, which is about three times higher than the kVCB dose but slightly lower than a single MV planar image dose (~2 cGy). For a low‐dose 3.4 MU MVCB body/pelvis scan with a half‐fan scan setting, the absolute imaging dose at the isocenter is approximately 3.4 MU×0.41cGy/MU=1.39 cGy, which is also about three times higher than the kVCB dose. However, depending on the kVCB technique used, a body scan or pelvis scan can result in lower dose using 2.5X MVCB; for example, for the pelvis spotlight technique in kVCB, the imaging dose can be as high as 2.22 cGy at isocenter and 6.28 cGy at 1 cm below surface. Furthermore, due to the MV energy range, the dose to bone is expected to be lower in MVCB than in kVCB and the MVCB dose can be easily incorporated into treatment plan optimization.^13^, ^19^

**Table 2:** Imaging dose of 2.5X MVCB and kVCB with a head and a body CTDI phantom. The nominal MVCB dose is calculated based on the low‐dose scan setting for a head phantom and pelvis phantom, at 2.4 MU and 3.4 MU, respectively

*Phantom/Scan Setting*	*POI*	*MVCB Dose (cGy/MU)*	*Nominal MVCB Dose (cGy)*	*kVCB Dose (cGy)*
Head,	Isocenter	0.56	1.34	0.39
full fan	1 cm below surface	0.73	1.75	0.60
Body,	Isocenter	0.41	1.39	0.37−2.22
half fan	1 cm below surface	0.42	1.43	0.54−6.28

## IV. DISCUSSION

Image‐guided applications have become a standard practice in radiotherapy in the recent years. With the increased use of kV X‐rays for patient setup and intrafractional motion management purposes, new treatment units often have fully integrated kV imaging systems for online imaging. On the other hand, almost all treatment units (either old or new models) are equipped with MV portal imager, but MV imaging is underutilized due to the poor image quality introduced by the high‐energy treatment beam (>6 MV). This study investigates the feasibility of MVCB with a prototype low‐dose 2.5 MV imaging system. With lower energy, the contrast produced by the 2.5 MV imaging system is improved compared to the 6 MV beam. However, the spatial resolution seems to suffer with smaller objects. This may be caused by the increased thickness of the scintillation material, although CsI has a columnar needle‐like crystal structure that acts as a light‐photon tunnel and minimizes dispersion.^16^, ^20^ Nonetheless, this observation does not transform into obvious effects such as blurring on phantom images, as seen in Figs. 4 to 8.

From the phantom studies, it was shown that the prototype 2.5 MV imaging system can provide better image quality with lower dose compared to 6X MVCB. This echoes the finding in the study by Beltran et al.,^(21)^ where they compared 4X MVCB with 6X MVCB. Ideally, comparison of image quality for 2.5 MV and 6 MV beams would be done at either identical doses or similar image quality (as measured by some standard parameters such as CNR, SNR, MTF). However, the ability to make exact comparisons was somewhat limited by constraints in the current version of the TrueBeam prototype control software, which limits control over several key parameters, such as number of projections and frame rate. A different approach was taken instead, where MVCB images were compared based on the minimum image quality for bony alignment. Intuitively, images were first generated with the lowest dose possible: each projection consists of multiple (integers of) beam pulses. With a dose per pulse of 0.0025 MU for the 2.5 MV beam, the lowest dose of a MVCB scan is approximately 1.1 MU, with an average of 450 projections for each MVCB scan. Similarly for the 6X beam, with a dose per pulse of 0.03 MU, the lowest dose of a MVCB scan is 13 MU (with ~450 projections). After several iterations with different imaging doses, it was found that the minimum dose to produce images with sufficient quality for bony alignment is 2−3 MU for 2.5X MVCB and 26 MU for 6X MVCB (i.e., 2−3 beam pulses per projection). In addition, for each phantom, 2.5X MVCB images were also acquired using a higher dose of approximately 9 MU to assess the extent to which image quality might be improved in terms of CNR.

It is undeniable that kVCB will always achieve better image quality than MVCB, owing to the laws of physics. The photoelectric effect that is responsible for the enhanced bone‐to‐tissue contrast in kV images is also the culprit for the prominent image artifacts for patients with metallic implants. With higher beam energy, MVCB is not affected by the high‐Z materials used in prosthesis. The minimization in metal artifacts can be very useful for treatment planning, as the streaking artifacts from high‐Z medium perturb the image quality of the surrounding regions in kV simulation images, especially in compact anatomical regions such as head and neck (with dental fillings). In addition, different from kV, MV images can directly yield true electron density values for purposes of inhomogeneity corrections in MV treatment planning.

## V. CONCLUSION

A prototype MV imaging system using unflattened 2.5 MV X‐rays with a thick CsI detector was tested. 2.5X MVCB scans were acquired and sufficient image quality for patient setup can be obtained with less than 3 MU. Overall, our results show that the prototype system is clinically feasible and may even have advantages over kVCB in some cases, such as imaging metallic objects. 2.5X MVCB is an economical alternative to kVCB, and provides better image quality than 6X with lower dose, making it particularly useful in developing countries. Continued development of the CsI detector, especially its control system, should lead to further improvements in image quality. Further developments for the image reconstruction are under progress, in particular with the use of noise filter and the optimization of the scatter correction and beam‐hardening correction.

## ACKNOWLEDGMENTS

The authors wish to thank Tom LoSasso and Michael Lovelock from MSKCC for their help and advice. The authors would also like to acknowledge Rene Hassanein, Luis Melo Carvalho, and Timo Berkus from Varian iLab for their help on image reconstruction. This project is supported in part by a Master Research Agreement from Varian Medical Systems.

## COPYRIGHT

This work is licensed under a Creative Commons Attribution 3.0 Unported License.
